# Assessing Heavy Episodic Drinking: A Random Survey of 18 to 34-Year-Olds in Four Cities in Four Different Continents

**DOI:** 10.3390/ijerph16050706

**Published:** 2019-02-27

**Authors:** Anne W. Taylor, Bridgette M. Bewick, Qian Ling, Valentina Kirzhanova, Paulo Alterwain, Eleonora Dal Grande, Graeme Tucker, Alfred B. Makanjuola

**Affiliations:** 1 Population Research & Outcome Studies, School of Medicine, The University of Adelaide, South Australia 5000, Australia; Eleonora.dalgrande@adelaide.edu.au (E.D.G.); Graeme.Tucker@adelaide.edu.au (G.T.); 2 School of Medicine, University of Leeds, Leeds LS29NL, UK; B.M.Bewick@leeds.ac.uk; 3 Chinese Center for Health Education, Beijing 100011, China; qianlingzh@126.com; 4 Department of Epidemiology, Federal Medical Research Centre for Psychiatry and Narcology, Ministry of Health of the Russian Federation, Moscow 101000, Russia; kirzhanovavv@mail.ru; 5 ProHumanitas Foundation, Montevideo 11300, Uruguay; alterw@adinet.com.uy; 6 Department of Behavioural Sciences, University of Ilorin Teaching Hospital, Ilorin 240242, Nigeria; makanju2@yahoo.com

**Keywords:** alcohol, adolescent, heavy episodic drinking, survey, Moscow, Montevideo, Wuhan, Ilorin

## Abstract

Background: Heavy episodic drinking (HED) can have health and social consequences. This study assesses the associations between HED and demographic, socioeconomic, motivation and effects indicators for people aged 18–34 years old living in four cities in different regions of the world. Method: Multistage random sampling was consistent across the four cities (Ilorin (Nigeria), Wuhan (China), Montevideo (Uruguay) and Moscow (Russia)). The questionnaire was forward/back translated and face-to-face interviewing was undertaken. A total of 6235 interviews were undertaken in 2014. Separate univariable and multivariable modelling was undertaken to determine the best predictors of HED. Results: HED prevalence was 9.0%. The best predictors differed for each city. The higher probability of HED in the final models included beliefs that they have reached adulthood, feeling relaxed as an effect of drinking alcohol, and forgetting problems as an effect of drinking alcohol. Lower probability of HED was associated with not being interested in alcohol as a reason for limiting alcohol, and the belief that drinking alcohol is too expensive or a waste of money. Conclusion: Although some indicators were common across the four cities, the variables included in the final models predominantly differed from city to city. The need for country-specific prevention and early intervention programs are warranted.

## 1. Introduction

Heavy episodic drinking (HED) of alcohol has a large effect on social, health and economic outcomes especially for adolescents and young adults. The consequences associated with this major public health concern in this younger population include behavioural (unsafe sex, driving while intoxicated, increased violence), physical and mental health (increased injuries, long term outcomes such as liver damage, depression, cognitive impairment, likelihood of long term alcohol dependency problems, alcohol poisoning, sexually transmitted diseases, and unintended pregnancies), and social issues (increased damage to property, increased violence, increased car accidents, and work problems) [1,2,3,4,5,6,7,8]. 

Considerable alcohol-related research has been undertaken on adolescents and young adults. This period of the lifespan corresponds with the initialization of lifestyle norms that are often maintained throughout the whole course of life. Studies have shown that problems associated with alcohol drinking in these formative years can result in alcohol-related drinking in later years [9,10,11]. It is also a time when social skills are developed and corresponds with important brain development [1,12]. Of concern is the research indicating that the traditional drinking patterns in this younger age group (such as drinking limited to social, family and cultural events) is being replaced by heavy binge drinking [13]. As such, research on this age group regarding HED during this major development period is seen as especially valuable.

Extensive demographic and socioeconomic descriptions and associations with other behaviours of those who undertake HED have been published [3,6,11,14,15]. Many of these studies in younger populations are limited to university/college students where HED is considered a major public health issue [1,7,8,16,17]. Among the college/university population, social reasons are the paramount reason for drinking alcohol and also one of the key indicators associated with HED [18,19].

The study undertaken here utilises a database collected from a population-based survey of 18 to 34-year-olds using a similar methodology from four cities on four different continents. As argued by Bloomfield et al., [20] international comparisons in aspects associated with alcohol consumption are not easy but this research addresses the need for more geographical based, methodologically-comparable studies especially in low- and middle-income countries [12,21,22,23,24]. In an endeavour to determine factors associated with HED, in conjunction with demographic and socio-economic status (SES) related questions, a range of beliefs, motivation and perception factors, known to be theoretically linked to drinking patterns and key determinants of alcohol consumption were included [19,25,26,27,28]. The definition of HED has changed over time and is inconsistently used across countries and studies, with other definitions often called binge drinking or risky single occasion drinking (RSOD) [6,7,17,29]. One of the most common definitions utilizing questions from the Alcohol Use Disorders Identification Test (AUDIT), defined as at least five drinks of alcohol in a single occasion, is used in this analysis [30,31]. 

## 2. Methods

Four cities were chosen pragmatically to be involved in the study based on diversity. The city of Wuhan, the capital of the Hubei Province in China; Moscow, the capital and the largest city of Russia; Ilorin which is the administrative capital of Kwara State, Nigeria; and Montevideo, the capital of Uruguay. Multistage random sampling was undertaken and was kept consistent across the four cities. In each randomly selected household, the person with the most recent birthday, aged between 18 and 34 years, and who had lived in the city for at least six months, was eligible and was invited to participate in the study. Data collection was interviewer-administered.

The questionnaire was forward-translated into the relevant languages (e.g., English to Chinese) and back-translated (e.g., Chinese to English) to ensure the questions were conceptually and culturally equivalent between the cities. A pilot study of 25 to 50 interviews was conducted in each city. The average length of the interviews was 15 minutes. Response rates ranged from 48.4% in Moscow to 95.0% in Ilorin.

In Wuhan, ethical approval was obtained from the Hubei Provincial CDC (Hubei Provincial Society for Health Promotion & Cigarette-smoking Control, HBPHP&CCS-2014-01), in Moscow from the Ethics Committee on the NRC on Addictions, in Ilorin from the Ethics Research Committee of the University of Ilorin (UERC/ASN/2014/007) and in Montevideo from the Pro Humanities Ethics Department. A more detailed methodology and demographic profile of respondents has previously been published [32].

### 2.1. Survey Questions

Respondents were asked (1) if they had ever consumed alcohol (excluding sips), (2) how often during the past 12 months had they drunk beer, wine, spirits (e.g., vodka, gin, whisky, brandy), and any other alcohol beverage, even in small amounts, and (3) during the past 12 months, how many alcoholic drinks had they had on a typical day when they drank alcohol. HED was determined by using the usual frequency of drinking (how often) and the usual number of drinks (how many) consumed per occasion with a person drinking at least five drinks at one occasion, at least once per month, deemed as having experienced a HED occasion.

Demographic questions included age (year of birth), sex, marital status (married, divorced/separated, widowed, never married), highest education obtained (secondary school or less; vocational/professional/non-university tertiary; university degree or higher), employment status (full time, part time, unemployed, homemaker, disabled and unable to work, other), religious affiliation (agnostic/atheist, Buddhist, Christian, Hindu, Jewish, Muslim, Sikh, another religion). Questions measuring the level of ‘adulthood’ assessed if the respondents thought they had reached adulthood and if they were financially independent and emotionally independent.

Respondents were asked context related questions including how often they drank when (1) out for an evening meal in a restaurant, (2) out for lunch at a restaurant, at bars, taverns or cocktail lounges, (3) spending time in someone else’s home, including parties, (4) spending a quiet evening at home, friends visited you in your home, (5) spending time with friends in a public place, such as a park, street or parking lot. Response categories were every day/nearly every day, at least once a week, at least once a month but less than once a week, at least once in the past 12 months but less than once a month, and never during the past 12 months. 

To determine the effects of alcohol, respondents were asked how true the following were when they drank alcohol: they (1) felt relaxed, (2) felt happy, (3) became aggressive towards other people, (4) felt more friendly and outgoing, (5) felt it easier to talk about feelings and problems, (6) forgot problems, (7) did something they later regretted, (8) felt sexual activity was more pleasurable, (9) felt more sexually attractive, (10) got into trouble with police, (11) had a lot of fun, (12) felt sick, (13) did not remember what happened/blacked out. Response categories were very often true, often true, sometimes true, rarely true, and never true.

Motivation for drinking alcohol included questions on how important the following reasons were for drinking alcohol: (1) to be sociable or polite (2) because others were drinking (3) to add to the enjoyment of the meals, (4) for health reasons, (5) to feel good, (7) to help in relaxation, (8) to forget worries, (9) to help feel less inhibited or shy, (9) to celebrate, (10) because of the taste, and (11) because of thirst. The response categories were very important, important, not very important, and not at all important.

Questions on reasons for limiting alcohol intake included the following: because (1) of a pregnancy or trying to become pregnant (females only), (2) of taste, (3) they did not like the effect it has, (4) they had seen bad examples of what alcohol could do, (5) they were previously hurt by somebody’s else drinking, (6) drinking could affect work or school performance, (7) drinking was too expensive or a waste of money, (8) of religious reasons, (9) they were brought up not to drink, (10) they had an alcohol problem or were afraid of becoming an alcoholic, (11) they were too young, (12) their friends and/or family members disapprove, (13) of health reasons, (14) they were just not interested. The possible responses were very important, important, not very important, not at all important. The responses were reverse coded to make interpretation of the models easier.

Perceptions about alcohol use included whether respondents agreed that (1) having a drink is one of the pleasures of life, (2) having a drink with someone is a way of being friendly, (3) there is nothing good to be said about drinking (strongly agree, agree, neither agree nor disagree, disagree, strongly disagree).

Respondents were also asked how many drinks people in certain situations should feel free to drink. The situations were the following: (1) as a mother, spending time with small children, (2) as a father, spending time with small children, (3) for a man out a bar with friends, (4) for a woman out at a bar with friends, (5) for a woman out with co-workers, (6) for a man out with co-workers, (7) for a man having dinner at home with a spouse or partner, (8) for a women having dinner at home with a spouse or partner. The responses options were 0 drinks, some drinking but not enough to feel the effects (1 or 2 drinks), enough to feel the effects but not become drunk, getting drunk is sometimes alright, and getting drunk is always alright.

### 2.2. Analysis

To reeducate or eliminate potential biases and to make sure that the results accurately reflect the population of interest, the data were weighted by age, sex and probability of selection. Data were analyzed using the complex samples procedures in Statistical Package for the Social Sciences (SPSS) version 20 for Windows (Chicago, IL, USA) to allow for the design effect of the sample. 

The analysis was undertaken by testing the association of the demographic, motivation, effect, perceptions and other variables listed above with HED, for those who had consumed alcohol in the past 12 months (n = 3645). Continuous variables were tested using a univariate logistic regression, and ordinal and categorical variables were tested using the chi-squared test. The birth year was re-centred to a range of 0 to 17 to produce more interpretable coefficients in the models and also to eliminate collinearity between the constant and birth year in the models. Any variable with a significant probability of <0.25 was included as a candidate predictor, as recommended by Hosmer and Lemeshow [33]. Continuous and ordinal variables were checked for linearity in the logit using the Box–Tidswell transformation and were included in the logistic regression models as continuous variables if linear in the logit. Candidate variables were not included in the analysis if they were ordinal in nature but not linear in the logit because the interpretation of ordinal variables is fraught when they are treated as categorical. Candidate variables were then removed sequentially if their significance probability was >0.05 in a backwards elimination of non-significant terms. Separate models were fitted at each step to account for the possibility of missing data. When a final model was produced, it was checked for confounding by comparing the coefficients estimated on the first model considered against the final model. The fit of the model was then tested using a Hosmer–Lemeshow test. Modelling was undertaken separately for each city.

## 3. Results

In total, n = 6235 interviews were undertaken (1391 in Ilorin, 1600 in Montevideo, 1604 in Moscow and 1640 in Wuhan). Males represented 47.6% of the sample in Ilorin, 49.2% in Montevideo, 49.2% in Moscow and 52.4% in Wuhan. The proportion of those who had consumed alcohol at all was 33.3% (95% CI 30.9–35.9) of respondents from Ilorin, 53.4% (95% CI 51.0–55.8) for Wuhan, 86.1% (95% CI 84.3–87.7) for Moscow, and 96.4% (95% CI 95.3–97.2) for Montevideo. 

The overall prevalence of HED at least once per month was 9.0% (Ilorin 3.2%; 95% CI 2.4–4.3, Montevideo 19.4%; 95% CI 17.5–21.4, Moscow 8.8%; 95% CI 7.5–10.3, Wuhan 3.9%; 95% CI 3.1–4.9). For each city, variation was observed in the prevalence of alcohol consumption at least once per week, twice a month or once a month, with Montevideo having the highest prevalence, and Ilorin and Wuhan exchanging the lowest prevalence rating depending upon the time-period measured (Table 1). A demographic summary of the sample by HED status is displayed in Table 2 with notable differences by most variables. The univariate responses to each variable for the four cities by HED status is included as the Supplementary Table S1.

Table 3 details the variables included in the final model for each city comprising of the best predictors of HED. The final logistic regression model for Ilorin (Table 4) indicated that the best predictors of HED were those who disagreed they were an adult (more likely to experience HED), those motivated against drinking because they are not interested (less likely), those who believe cost is important (less likely), those who frequently have a quiet drink at home (more likely), with birth year being a confounder and a significant predictor (with younger respondents less likely to experience HED). Included in the final logistic regression model for Montevideo were those who disagreed that they were an adult (more likely to experience HED), those who often feel relaxed when they drink (more likely), those who frequently have a quiet drink at home (more likely), those who frequently drink with friends in public places (more likely), and males (more likely). Gender was a significant predictor. Included in the final logistic regression model for Moscow were those who often get aggressive when drinking (more likely), those who often forget their problems when they drink (more likely), those who think it is right for a Father spending time with small children to drink a lot (more likely), and males (more likely). Gender was a significant predictor. Included in the final logistic regression model for Wuhan was those who were employed (more likely), those who often become aggressive when drinking (more likely), and those motivated against drinking because they are not interested (less likely). Birth year was a confounder but not a significant predictor. Employment status was a significant predictor.

## 4. Discussion

This analysis has provided multivariable models displaying variables that are relevant to the HED of alcohol in the 18- to 34-year-olds in each of the cities we studied. Not one of the variables examined was found in all cities highlighting the different, independent and unique values, and beliefs and behaviours in each city. Different alcohol consumption patterns are a reflection of a range of concepts including traditions, the influence of different official government policies and legislation in each region/country, as well as religion, income, occupation, gender and associated behaviours, beliefs and motivations of each individual [20,24,26,28,34]. The final HED models provided in the four cities studied in this research highlight some of these differences.

Our prevalence rates of HED (except for Montevideo) are relatively low although definitional issues result in comparative problems. The analysis of USA data by Patrick et al (2017) [35] provides the overall HED prevalence rates (5+ drinks in the past 2 weeks) ranging from 17.7% for 18-year-olds to 25% for 29- to 30-year-olds with a peak in the 21 to 26-year-olds of over 32%. In this Patrick et al study [35] rates declined from 2005-2015 for all age groups except for 27- to 30-year-olds. Perhaps the good news in HED research is the evidence of some declining prevalence rates in these younger age groups [35,36] at least in the USA, although the rates are still relatively high and outcomes alarming and costly. Very little other international comparisons, using random non-university based populations, are available for the cities/countries and age range we studied.

As reported by others, family, friends, and important social contacts have a significant influence on alcohol drinking behaviours and patterns especially HED [6,17,26,27,37,38,39]. The social influence is especially important in the age groups we were studying [26]. Only one of the final models (Montevideo) highlighted the importance of social occasions with those more likely to experience HED reporting drinking alcohol when ‘spending time with friends in a public place such as a park, street or parking lot’. Studies have shown that peer pressure is more likely to come from same-sex friends although a partner influence is also important [26,28]. This partner influence was not shown in our study in any of the cities. In addition, none of the other family/friend variables, such as being a reason for limiting alcohol (e.g., the way they were brought up, family disapproval) or being a reason for drinking (e.g., others drinking, to be social, polite), were included in any model. Factors that might explain why these variables were not included in the final models include the fact that our study sample was not sourced from college or university students and the fact that we assessed the wider age range from 18 to 35-year-olds. As highlighted by other research, many drinkers stop being ‘peer-influenced’ in their drinking habits as they reach early adulthood and did not need social occasions to consume high levels of alcohol [19,40].

In contrast, a variable that was incorporated into the final models for HED for Ilorin and Montevideo was having alcohol during quiet evenings at home. Alcohol consumption worldwide has long been associated with ‘hospitality and ritual’ [41]. This is especially the case in Nigeria with traditional practices coupled with religious protocols dominating alcohol consumption patterns [42,43,44]. Notwithstanding, Obot [44] highlighted the importance and increasing relevance of ‘drinking at home’ that has emerged in Nigeria in recent times which is now as important as the traditional cultural occasions. This is also typically occurring across many African countries [43]. With the commercialization of alcohol and different contexts being associated with consumption trends, changes in behaviours have taken place [42]. In the work by Ibanga et al. [42], drinking in the home was the second most preferred place to consume alcohol (behind ‘at a bar’) on a daily basis in a sample of Nigerian adults. This response was the most preferred for weekly drinkers. Montevideo’s inclusion of this variable may be associated with their ‘drinking with meals’ concept, with Montevideo’s drinking culture often compared to the Southern European ‘wine with meals’ habit. 

The cost was a protective determinate for HED for Ilorin respondents, with the variable that assessed the value that drinking alcohol was ‘too expensive and a waste of money’ being associated with lower levels of HED. Country-wide economic indicators such as GDP have been related to alcohol consumption patterns and outcomes [39] with higher GDP being associated with increased alcohol consumption and HED [16]. Alcohol consumption in Nigeria is low overall but for those who do consume alcohol, large amounts are common [42,43,44,45].

Previous research has indicated that HED is usually associated with males [1,17,29,34] and our study also indicated that males in Montevideo and Moscow, both high overall alcohol consumption cities, are more likely to experience HED sessions. Other studies have shown the alarming increased trends of alcohol consumption and HED in younger females [26,34,46]. In fact, increases for females have been faster than for males in recent years [3,23,36,47].

In terms of motivation and effects of when they drink alcohol, diametrically opposite results were found. There was a belief in Montevideo that their high alcohol consumption was going to result in a feeling of ‘being relaxed’. Moscow HED drinkers also provided an expectation of the effect of being able to ‘forget problems’. This ‘forget problems’ explanation is often classified in the drinking alcohol to cope with motivation criteria [48]. Conversely, the Moscow and Wuhan perceptions were that HED would result in them becoming ‘more aggressive’—a behaviour that would not necessarily be desirable. Previous research has shown that HED is associated with increased aggression and fighting [5]. This compounding of alcohol with violence has major public health and safety consequences in adolescents and young adults, and a focus of many alcohol prevention programs worldwide in these younger age groups [49,50].

Of interest was the range of variables, previously cited by researchers as important HED indicators, which did not feature in any of the final models. These included marital status, which has been found to be an important variable associated with higher rates of HED in major international studies [26,38]. The role of religion in research into alcohol consumption has shown strong associations and interestingly, in this multinational study, religion was not a factor in any of the final models. 

Weaknesses associated with this study include the fact that this analysis is limited to a cross-sectional study with no cause or effect or long-term trends in transition implied. Furthermore, no gender-specific analysis was undertaken to determine differences, although gender was included in all multivariate analyses. There was no assessment of a measure of acceptance within each city on the satisfactory levels and tolerance of intoxication. Due to the analysis undertaken, we did not incorporate how often, how regular, where, and when alcohol consumption occurred which are all recognized as important determinants of alcohol consumption [24]. We also acknowledge that the different countries have specific legislation and we did not take into account the different legal age limits associated with alcohol consumption. City-specific details and associations will be the responsibility of the researchers from the organizations in each city responsible for the data collection.

In addition, we assessed respondents who had consumed alcohol in the past 12 months with wide variations in means and mediums reported for each city. Different results may have been found if only regular drinkers were assessed. It was outside of the remit of the current research to include comparisons with other behaviours (e.g., drugs, cigarettes) which have been shown to be highly correlated in HEDs [24]. No economic comparisons were undertaken and HED is known to be more common in wealthier counties [14,26]. A meaningful SES and economic variable allowing computation of an economic/SES variable across the cities would be advantageous. Future publications may incorporate some of these additional features. It must also be acknowledged that bias could result in the cultural differences in responding to the questions asked in this survey. As argued by Graham et al. [24], culture affects the different experiences associated with drinking alcohol, as well as the acceptance of reporting negative aspects. We sought to overcome this possible weakness with rigid pilot testing and the use, as much as possible, of valid and reliable questions. In addition, the relationship between HED and Blood Alcohol Content (BAC) was not included in the analysis as the relationship is complex with BAC differing by weight, sex, and consumption context (e.g., food, the speed of drinking, non-alcoholic drinks). 

The strengths of this study include the diversity of the cities studied, the focus on the limited age range, the relatively large sample size, the high response rates and the use of a probability-based sampling methodology (stratified, clustered, systematic) in each city. The weighting of the data allowed for estimates to be representative of the general population. A further strength was the use, as far as possible, of comparable measures of alcohol consumption and demographic variables and similar methodological aspects such as sample selection, protocols, and administration. This included city specific interviewing. Additionally, the involvement of local communities and language-specific interviewing with translation and back translation of all questionnaires are seen as strengths of the study. Furthermore, the use of a pre-designed epidemiological-sound methodology, rather than post-data collection manipulation of already collected data, is seen as an additional strength. Extensive research investigating alcohol consumption of young people in the European Union and North America has been undertaken and therefore a strength of the current study is its investigation of a population where there is a relative paucity of research.

## 5. Conclusions

Addressing some of the shortfalls in the social and health consequences associated with HED is an important research endeavour. Very few benefits are associated with HED but the negative outcomes are well documented. As such, this major worldwide public health issue requires primary prevention activities that are country- and culturally-specific but address global and multicultural issues so that HED does not continue to be problematic with some tragic outcomes. The interesting declining trend of alcohol consumption reported in Sweden for youth that can be related to the increase in prevention activities and programs aimed at youth [51] might provide some ways for forward endeavours. Other typical measures cited to overcome problem alcohol drinking such as raising taxes [1], increased legal age [1,13], increased cost [4,52] restricted availability [4], and restrictions on advertising and promotion [4,7], are also warranted in an endeavour to overcome poor HED outcomes in this priority age group. This analysis has highlighted factors that may assist in providing prevention and early intervention ideas in addressing this worldwide and multiculturally complex public health problem. 

## Figures and Tables

**Table 1:** Heavy episodic drinking five or more drinks for three levels of frequency of heavy drinking (at least once a week, at least twice a month at least once a month) by country.

	Ilorin, Nigeria		Montevideo, Uruguay		Moscow, Russia		Wuhan, China	
	n	% (95% CI)	N	% (95% CI)	n	% (95% CI)	n	% (95% CI)
At least once a week	41	2.9 (2.2–4.0) *	203	12.7 (11.2–14.4) *	68	4.2 (3.4–5.3) *	26	1.6 (1.1–2.3) *
At least twice a month	42	3.1 (2.3–4.1) *	273	17.1 (15.3–19.0) *	112	7.0 (5.9–8.4)	50	3.0 (2.3–4.0) *
At least once a month	44	3.2 (2.4–4.3) *	310	19.4 (17.5–21.4) *	140	8.8 (7.5–10.3)	64	3.9 (3.1–4.9) *

CI: Confidence Interval. Note: The weighting of data can result in rounding discrepancies or totals not adding. Do not know/refused not included. * Significantly different to all other categories combined (*p* < 0.05).

**Table 2:** The proportion of heavy episodic drinkers by the demographic profile of respondents.

	Ilorin Nigeria		Montevideo Uruguay		Moscow Russia		Wuhan China		Overall	
	HED (yes)		HED (yes)		HED (yes)		HED (yes)		HED (yes)	
	N	%	N	%	N	%	N	%	N	%
***Overall***	***1393***		***1600***		***1593***		***1635***		***6221***	
**SEX**										
Male	33	73.3	236	75.9	110	78.6	38	59.4	417	74.5
Female	12	26.7	75	24.1	30	21.4	26	40.6	143	25.5
**YOU HAVE REACHED ADULTHOOD**										
Strongly agree	21	46.7	52	16.9	109	77.9	45	70.3	227	40.8
Agree	16	35.6	99	32.1	28	20.0	18	28.1	161	28.9
Neither agree nor disagree	1	2.2	51	16.6	3	2.1	1	1.6	56	10.1
Disagree	4	8.9	93	30.2	0	0.0	0	0.0	97	17.4
Strongly disagree	3	6.7	13	4.2	0	0.0	0	0.0	16	2.9
**FINANCIALLY INDEPENDENT OF PARENTS OR OTHER FAMILY MEMBERS**										
Strongly agree	9	20.5	71	22.9	78	57.4	32	49.2	190	34.2
Agree	15	34.1	107	34.5	29	21.3	25	38.5	176	31.7
Neither agree nor disagree	4	9.1	36	11.6	19	14.0	4	6.2	63	11.4
Disagree	7	15.9	74	23.9	9	6.6	4	6.2	94	16.9
Strongly disagree	9	20.5	22	7.1	1	0.7	0	0.0	32	5.8
**EMOTIONALLY INDEPENDENT OF YOUR PARENTS OR GUARDIANS**										
Strongly agree	8	17.8	69	22.3	63	46.3	33	52.4	173	31.2
Agree	11	24.4	153	49.4	33	24.3	21	33.3	218	39.4
Neither agree nor disagree	2	4.4	28	9.0	25	18.4	4	6.3	59	10.6
Disagree	15	33.3	47	15.2	13	9.6	5	7.9	80	14.4
Strongly disagree	9	20.0	13	4.2	2	1.5	0	0.0	24	4.3
**HIGHEST LEVEL OF EDUCATION COMPLETED**										
Secondary school or less	23	51.1	205	65.9	20	14.3	19	29.7	267	47.7
Vocational/professional/Non-university tertiary education	9	20.0	90	28.9	62	44.3	40	62.5	201	35.9
University degree or higher	13	28.9	16	5.1	58	41.4	5	7.8	92	16.4
**EMPLOYMENT SITUATION**										
Employed full-time	25	55.6	167	53.7	107	76.4	51	78.5	350	62.4
Employed part-time	5	11.1	46	14.8	13	9.3	4	6.2	68	12.1
Unemployed	8	17.8	58	18.6	4	2.9	0	0.0	70	12.5
Student	7	15.6	36	11.6	0	0.0	7	10.8	50	8.9
Other	0	0.0	4	1.3	16	11.4	3	4.6	23	4.1
**RELIGIOUS AFFILIATION**										0
Agnostic/atheist	0	0.0	168	54.2	22	15.7	57	87.7	247	44.2
Christian	32	72.7	128	41.3	107	76.4	1	1.5	268	47.9
Muslim	12	27.3	0	0.0	3	2.1	0	0.0	15	2.7
Other	0	0.0	13	4.2	2	1.4	1	1.5	16	2.9
**MARTIAL STATUS**										
Married	13	28.9	112	36.1	50	35.7	35	54.7	210	37.6
Divorced or separated/Widowed	4	8.9	13	4.2	16	11.4	1	1.6	34	6.1
Never married	28	62.2	185	59.7	74	52.9	28	43.8	315	56.4

**Table 3:** The univariable associations of heavy episodic drinkers determined by city, country.

Ilorin, Nigeria			Montevideo, Uruguay			Moscow, Russia			Wuhan, China		
	N	%		N	%		N	%		N	%
Reasons for limiting or not drinking alcohol: Not interested									Reasons for limiting or not drinking alcohol: Not interested		
Not at all important	111	40.8							Not at all important	152	22.7
Not very important	33	12.1							Not very important	264	39.4
Important	54	20.0							Important	142	21.2
Very important	74	27.2							Very important	113	16.8
Reasons for limiting or not drinking alcohol: Cost											
Not at all important	167	61.0									
Not very important	83	30.2									
Important	18	6.4									
Very important	7	2.4									
Frequency of alcohol consumption: Quiet evening at home			Frequency of alcohol consumption: Quiet evening at home								
Never during the past 12 months	158	57.5	Never during the past 12 months	730	54.0						
At least 1/12 mths to <1/mth	22	8.1	At least 1/12 mths to <1/mth	205	15.2						
At least 1/month to <1/week	37	13.6	At least 1/month to <1/week	211	15.6						
At least 1/week	42	15.3	At least 1/week	178	13.2						
Nearly/Every day	15	5.5	Nearly/Every day	28	2.1						
			Frequency of alcohol consumption: Public place with friends								
			Never during the past 12 months	789	58.4						
			At least 1/12 mths to <1/mth	216	16.0						
			At least 1/month to <1/week	190	14.1						
			At least 1/week	136	10.1						
			Nearly/Every day	19	1.4						
						Effects of drinking alcohol: Aggressive			Effects of drinking alcohol: Aggressive		
						Never true	731	62.1	Never true	428	63.5
						Rarely true	255	21.7	Rarely true	188	27.9
						Sometimes true	126	10.7	Sometimes true	49	7.3
						Often true	44	3.7	Often true	8	1.1
						Very Often true	20	1.7	Very Often true	1	0.2
			Effects of drinking alcohol: Relaxed								
			Never true	226	16.1						
			Rarely true	237	16.9						
			Sometimes true	390	28.8						
			Often true	254	18.7						
			Very often true	248	18.3						
						Effects of drinking alcohol: Forget problems					
						Never true	161	13.5			
						Rarely true	198	16.5			
						Sometimes true	371	31.1			
						Often true	312	26.1			
						Very often true	153	12.8			
						Opinion on how much this person should drink:Father with small children					
						0 drinks	932	76.3			
						Some drinking (1–2 drinks)	251	20.5			
						Feel effects but not drunk	28	2.3			
						Drunk sometimes alright	4	0.3			
						Getting drunk always alright	7	0.6			
You have reached adulthood			You have reached adulthood								
Strongly agree	166	59.0	Strongly agree	306	21.8						
Agree	68	24.3	Agree	572	40.8						
Neither agree nor disagree	16	5.9	Neither agree nor disagree	189	13.5						
Disagree	27	9.8	Disagree	292	20.8						
Strongly disagree	3	1.0	Strongly disagree	42	3.0						
									Employment situation		
									Not employed	227	30.4
									Employed	520	69.6
			Gender			Gender					
			Male	710	50.6	Male	637	51.6			
			Female	693	49.4	Female	598	48.4			

**Table 4:** The multivariable associations of heavy episodic drinkers by the city, country.

Ilorin, Nigeria				Montevideo, Uruguay				Moscow, Russia				Wuhan, China			
	OR (95% OR)	Adj p	Unadj p		OR (95% OR)	Adj p	Unadj p		OR (95% OR)	Adj p	Unadj p		OR (95% OR)	Adj p	Unadj p
You have reached adulthood	2.18 (1.51–3.16)	0.012	0.170	You have reached adulthood	1.39 (1.28–1.50)	<0.001	<0.001								
								Effects of drinking alcohol: Aggressive	1.41 (1.13–1.76)	0.003	<0.001	Effects of drinking alcohol: Aggressive	1.63 (1.18–2.25)	0.003	0.004
Reasons limiting/not drinking alcohol:Not interested	0.77 (0.65–0.91)	0.022	0.029									Reasons limiting or not drinking alcohol: Not interested	0.58 (0.40–0.85)	0.006	0.002
				Effects of drinking alcohol: Relaxed	1.27 (1.15–1.41)	<0.001	<0.001								
								Effects of drinking alcohol: Forget problems	1.60 (1.29–1.99)	<0.001	<0.001				
Reasons limiting or not drinking alcohol: Cost	0.33 (0.20–0.55)	0.012	0.013												
Specific situationsQuiet evening at home	1.97 (1.23–3.16)	0.025	<0.001	Specific situations: Quiet evening at home	1.27 (1.17–1.39)	<0.001	<0.001								
								Opinion on amount: Father with small children	1.84 (1.35–2.50)	<0.001	<0.001				
												Employment situation	2.51 (1.23–5.13)	0.004	0.004
				Specific situationsPublic place with friends	1.63 (1.41–1.88)	<0.001	<0.001								
				Gender	0.30 (0.21–0.42)	<0.001	<0.001	Gender	0.35 (0.21–0.59)	<0.001	<0.001				

## Supplementary Materials

The following are available online at https://www.mdpi.com/1660-4601/16/5/706/s1, Table S1: Proportion of heavy episodic drinkers by various statements on drinking alcohol.
