# Supplementary material for: Assessing Heavy Episodic Drinking: A Random Survey of 18 to 34-Year-Olds in Four Cities in Four Different Continents
Source: Int J Environ Res Public Health. 2019 Feb 27;16(5):706. doi: 10.3390/ijerph16050706 (PMC6427135; doi:10.3390/ijerph16050706)
Supplement: Supplementary file 1 [file ijerph-16-00706-s001.pdf]

Supplementary Table 1: Proportion of heavy episodic drinkers by various statements on drinking alcohol

	Ilorin (Nigeria)				Montevideo (Uruguay)				Moscow (Russia)				Wuhan (China)			
	Non-HED		HED		Non-HED		HED		Non-HED		HED		Non-HED		HED	
	N	%	N	%	N	%	N	%	N	%	N	%	N	%	N	%
**Effects of drinking alcohol**																
**RELAXED**																
Very often true	111	47.6	22	50.0	172	16.5	76	24.4	256	24.0	65	46.4	49	7.9	8	12.5
Often true	55	23.6	16	36.4	170	16.3	84	27.0	417	39.0	56	40.0	230	37.2	26	40.6
Sometimes true	34	14.6	4	9.1	302	28.9	88	28.3	301	28.2	12	8.6	235	38.0	22	34.4
Rarely true	21	9.0	1	2.3	202	19.3	35	11.3	74	6.9	7	5	56	9.1	4	6.3
Never true	12	5.2	1	2.3	198	19.0	28	9	20	1.9	-	-	48	7.8	4	6.3
**HAPPY**																
Very often true	100	43.3	17	38.6	159	15.3	88	28.4	152	14.6	47	34.8	48	7.8	6	9.4
Often true	44	19	17	38.6	200	19.2	92	29.7	299	28.7	40	29.6	216	35.1	24	37.5
Sometimes true	51	22.1	5	11.4	297	28.5	71	22.9	363	34.9	32	23.7	217	35.3	23	35.9
Rarely true	22	9.5	4	9.1	168	16.1	29	9.4	141	13.6	11	8.1	81	13.2	7	10.9
Never true	14	6.1	1	2.3	218	20.9	30	9.7	85	8.2	5	3.7	53	8.6	4	6.3
**AGGRESSIVE**																
Very often true	1	0.4	-	-	9	0.9	14	4.5	13	1.3	7	5.1	1	0.2	-	-
Often true	7	3.0	3	6.8	14	1.3	12	3.9	29	2.8	14	10.3	5	0.8	1	1.6
Sometimes true	23	9.9	3	6.8	43	4.1	22	7.1	91	8.8	34	25.0	42	6.9	7	11.5
Rarely true	45	19.4	4	9.1	103	9.9	54	17.4	214	20.7	41	30.1	161	26.4	27	44.3
Never true	156	67.2	34	77.3	874	83.8	208	67.1	686	66.4	40	29.4	400	65.7	26	42.6

Supplementary Table 1: Proportion of heavy episodic drinkers by various statements on drinking alcohol (Cont.)

	Ilorin (Nigeria)				Montevideo (Uruguay)				Moscow (Russia)				Wuhan (China)			
	Non-HED		HED		Non-HED		HED		Non-HED		HED		Non-HED		HED	
	N	%	N	%	N	%	N	%	N	%	N	%	N	%	N	%
**FRIENDLY**																
Very often true	33	14.3	7	15.9	110	10.6	45	14.6	182	17.5	34	24.8	31	5.1	3	4.8
Often true	69	30.0	10	22.7	144	13.9	74	24.0	363	34.9	51	37.2	131	21.4	26	41.3
Sometimes true	52	22.6	9	20.5	278	26.8	100	32.5	350	33.7	41	29.9	218	35.6	12	19.0
Rarely true	17	7.4	10	22.7	178	17.1	44	14.3	100	9.6	7	5.1	142	23.2	13	20.6
Never true	59	25.7	8	18.2	328	31.6	45	14.6	45	4.3	4	2.9	90	14.7	9	14.3
**EASIER TO TALK**																
Very often true	19	8.2	9	20.0	90	8.6	41	13.2	130	12.4	38	27.7	24	3.9	2	3.1
Often true	24	10.3	9	20.0	124	11.9	54	17.4	338	32.3	56	40.9	98	16.0	22	34.4
Sometimes true	69	29.7	10	22.2	204	19.6	73	23.5	340	32.5	32	23.4	182	29.7	17	26.6
Rarely true	41	17.7	4	8.9	161	15.5	54	17.4	144	13.8	9	6.6	154	25.1	15	23.4
Never true	79	34.1	13	28.9	462	44.4	88	28.4	95	9.1	2	1.5	155	25.3	8	12.5
**FORGET PROBLEMS**																
Very often true	16	6.9	6	14.0	67	6.4	27	8.7	107	10.2	45	32.6	23	3.7	1	1.5
Often true	21	9.1	7	16.3	68	6.5	58	18.6	265	25.4	43	31.2	91	14.8	20	30.8
Sometimes true	51	22.0	9	20.9	146	14.0	58	18.6	335	32.1	32	23.2	158	25.6	20	30.8
Rarely true	43	18.5	7	16.3	172	16.5	45	14.5	183	17.5	13	9.4	173	28.1	14	21.5
Never true	101	43.5	14	32.6	587	56.4	123	39.5	155	14.8	5	3.6	171	27.8	10	15.4

Supplementary Table 1: Proportion of heavy episodic drinkers by various statements on drinking alcohol (Cont.)

	Ilorin (Nigeria)				Montevideo (Uruguay)				Moscow (Russia)				Wuhan (China)			
	Non-HED		HED		Non-HED		HED		Non-HED		HED		Non-HED		HED	
	N	%	N	%	N	%	N	%	N	%	N	%	N	%	N	%
**REGRET**																
Very often true	5	2.2	-	-	18	1.7	7	2.3	31	3.0	8	5.9	2	0.3	-	-
Often true	7	3.0	1	2.3	29	2.8	19	6.1	60	5.8	21	15.4	16	2.6	2	3.3
Sometimes true	16	6.9	7	15.9	80	7.7	55	17.7	144	14.0	45	33.1	42	6.9	8	13.3
Rarely true	27	11.6	1	2.3	154	14.8	53	17.1	271	26.4	38	27.9	140	23.1	18	30.0
Never true	177	76.3	35	79.5	763	73.1	176	56.8	522	50.8	24	17.6	406	67.0	32	53.3
**SEX MORE PLEASURABLE**																
Very often true	35	16.0	7	15.9	45	4.4	27	8.8	44	5.3	18	14.9	8	1.9	1	2.0
Often true	41	18.7	7	15.9	38	3.7	29	9.4	142	17.0	37	30.6	36	8.6	4	8.0
Sometimes true	45	20.5	11	25.0	154	15.0	81	26.3	260	31.2	28	23.1	81	19.3	12	24.0
Rarely true	28	12.8	3	6.8	129	12.5	40	13.0	171	20.5	22	18.2	95	22.6	12	24.0
Never true	70	32	16	36.4	662	64.4	131	42.5	216	25.9	16	13.2	200	47.6	21	42.0
**FEEL MORE ATTRACTIVE**																
Very often true	31	14.1	8	18.6	30	2.9	14	4.5	88	10.0	32	25.0	5	1.1	1	1.9
Often true	39	17.7	9	20.9	26	2.5	19	6.2	183	20.7	23	18.0	27	5.7	3	5.8
Sometimes true	36	16.4	10	23.3	104	10.0	61	19.8	292	33.1	41	32.0	69	14.6	8	15.4
Rarely true	31	14.1	4	9.3	128	12.3	47	15.3	145	16.4	20	15.6	101	21.4	11	21.2
Never true	83	37.7	12	27.9	749	72.2	167	54.2	174	19.7	12	9.4	270	57.2	29	55.8

Supplementary Table 1: Proportion of heavy episodic drinkers by various statements on drinking alcohol (Cont.)

	Ilorin (Nigeria)				Montevideo (Uruguay)				Moscow (Russia)				Wuhan (China)			
	Non-HED		HED		Non-HED		HED		Non-HED		HED		Non-HED		HED	
	N	%	N	%	N	%	N	%	N	%	N	%	N	%	N	%
**TROUBLE WITH POLICE**																
Very often true	-	-	-	-	4	0.4	4	1.3	7	0.7	-	-	-	-	-	-
Often true	4	1.7	-	-	9	0.9	5	1.6	13	1.3	-	-	4	0.7	-	-
Sometimes true	3	1.3	-	-	17	1.6	17	5.5	42	4.0	19	13.9	18	3.0	1	1.8
Rarely true	18	7.8	5	11.4	48	4.6	25	8.1	99	9.5	27	19.7	38	6.4	8	14.0
Never true	205	89.1	39	88.6	967	92.5	259	83.5	877	84.5	91	66.4	535	89.9	48	84.2
**FUN**																
Very often true	84	36.2	11	24.4	300	28.8	153	49.7	169	15.8	43	30.9	11	1.8	1	1.6
Often true	60	25.9	10	22.2	281	27.0	94	30.5	344	32.2	52	37.4	79	13.1	14	22.2
Sometimes true	47	20.3	13	28.9	231	22.2	46	14.9	341	32.0	33	23.7	217	36.1	16	25.4
Rarely true	13	5.6	4	8.9	85	8.2	8	2.6	131	12.3	6	4.3	149	24.8	18	28.6
Never true	28	12.1	7	15.6	144	13.8	7	2.3	82	7.7	5	3.6	145	24.1	14	22.2
**BLACK OUT**																
Very often true	3	1.3	-	-	13	1.2	5	1.6	14	1.3	1	0.7	9	1.5	-	-
Often true	3	1.3	-	-	14	1.3	13	4.2	37	3.5	15	10.9	24	4.0	3	4.7
Sometimes true	14	6.0	3	7.0	55	5.3	54	17.4	65	6.2	27	19.7	97	16.1	13	20.3
Rarely true	20	8.6	6	14.0	156	14.9	65	20.9	192	18.2	52	38.0	141	23.3	24	37.5
Never true	3	1.3	-	-	13	1.2	5	1.6	14	1.3	1	0.7	9	1.5	-	-

Supplementary Table 1: Proportion of heavy episodic drinkers by various statements on drinking alcohol (Cont.)

	Ilorin (Nigeria)				Montevideo (Uruguay)				Moscow (Russia)				Wuhan (China)			
	Non-HED		HED		Non-HED		HED		Non-HED		HED		Non-HED		HED	
	N	%	N	%	N	%	N	%	N	%	N	%	N	%	N	%
Importance of the following reasons for drinking alcohol																
**HEALTH**																
Very important	11	4.8	4	9.1	19	1.8	9	2.9	103	10.0	19	14.2	71	11.7	2	3.2
Important	23	10.0	13	29.5	94	9.0	14	4.5	257	24.9	34	25.4	214	35.1	7	11.1
Not very important	19	8.3	10	22.7	151	14.5	43	14.0	314	30.4	44	32.8	185	30.4	36	57.1
Not at all important	176	76.9	17	38.6	779	74.7	242	78.6	359	34.8	37	27.6	139	22.8	18	28.6
**FEEL GOOD**																
Very important	90	38.6	15	34.1	35	3.3	27	8.7	160	15.3	23	17.0	32	5.2	5	7.9
Important	91	39.1	23	52.3	216	20.7	107	34.6	411	39.2	63	46.7	198	32.0	16	25.4
Not very important	31	13.3	5	11.4	313	30.0	88	28.5	266	25.4	30	22.2	252	40.8	34	54.0
Not at all important	21	9.0	1	2.3	481	46.0	87	28.2	211	20.1	19	14.1	136	22	8	12.7
**FORGET WORRIES**																
Very important	21	9.2	5	11.4	24	2.3	12	3.9	172	16.3	43	31.4	24	3.9	2	3.1
Important	32	14.0	14	31.8	124	11.9	90	29.0	405	38.3	61	44.5	141	23.0	18	28.1
Not very important	76	33.3	8	18.2	240	23.1	73	23.5	294	27.8	26	19.0	248	40.4	31	48.4
Not at all important	99	43.4	17	38.6	652	62.7	135	43.5	187	17.7	7	5.1	201	32.7	13	20.3

Supplementary Table 1: Proportion of heavy episodic drinkers by various statements on drinking alcohol (Cont.)

	Ilorin (Nigeria)				Montevideo (Uruguay)				Moscow (Russia)				Wuhan (China)			
	Non-HED		HED		Non-HED		HED		Non-HED		HED		Non-HED		HED	
	N	%	N	%	N	%	N	%	N	%	N	%	N	%	N	%
**INHIBITION**																
Very important	17	7.5	4	9.3	32	3.1	22	7.2	129	12.2	27	19.6	30	4.9	3	4.6
Important	38	16.7	3	7.0	182	17.5	83	27.0	349	33.0	58	42.0	104	16.9	9	13.8
Not very important	62	27.2	14	32.6	244	23.4	76	24.8	305	28.8	36	26.1	269	43.8	37	56.9
Not at all important	111	48.7	22	51.2	584	56.0	126	41.0	275	26.0	17	12.3	211	34.4	16	24.6
**CELEBRATE**																
Very important	144	62.3	26	57.8	362	34.7	162	52.4	503	47.1	68	49.6	119	19.0	15	23.4
Important	65	28.1	15	33.3	469	45.0	121	39.2	442	41.3	49	35.8	363	58.0	36	56.3
Not very important	12	5.2	1	2.2	151	14.5	13	4.2	92	8.6	16	11.7	99	15.8	10	15.6
Not at all important	10	4.3	3	6.7	61	5.8	13	4.2	32	3.0	4	2.9	45	7.2	3	4.7
**TASTE**																
Very important	3	1.3	3	6.8	115	11.0	57	18.4	249	23.5	44	32.4	17	2.8	-	-
Important	24	10.4	4	9.1	420	40.3	153	49.4	478	45.2	47	34.6	81	13.3	6	9.4
Not very important	27	11.7	10	22.7	225	21.6	49	15.8	224	21.2	36	26.5	244	40.0	30	46.9
Not at all important	176	76.5	27	61.4	283	27.1	51	16.5	107	10.1	9	6.6	268	43.9	28	43.8
**THIRST**																
Very important	3	1.3	-	-	26	2.5	18	5.8	97	9.3	19	13.9	4	0.7	1	1.6
Important	26	11.4	6	14.0	95	9.1	61	19.7	279	26.7	47	34.3	18	3.0	1	1.6
Not very important	18	7.9	8	18.6	168	16.1	66	21.4	313	30.0	43	31.4	184	30.4	18	28.1
Not at all important	181	79.4	29	67.4	756	72.3	164	53.1	354	33.9	28	20.4	400	66.0	44	68.8

Supplementary Table 1: Proportion of heavy episodic drinkers by various statements on drinking alcohol (Cont.)

	Ilorin (Nigeria)				Montevideo (Uruguay)				Moscow (Russia)				Wuhan (China)			
	Non-HED		HED		Non-HED		HED		Non-HED		HED		Non-HED		HED	
	N	%	N	%	N	%	N	%	N	%	N	%	N	%	N	%
Importance of the following reasons for limiting or not drinking alcohol at all																
**PREGNANT**																
Very important	8	10.8	-	-	415	71.1	47	64.4	426	78.6	20	71.4	95	85.6	-	-
Important	10	13.5	-	-	69	11.8	12	16.4	96	17.7	5	17.9	12	10.8	-	-
Not very important	5	6.8	4	36.4	23	3.9	8	11.0	11	2.0	1	3.6	3	2.7	-	-
Not at all important	51	68.9	7	63.6	77	13.2	6	8.2	9	1.7	2	7.1	1	0.9	-	-
**TASTE**																
Very important	5	2.2	-	-	200	19.3	45	14.5	315	30.3	23	16.8	48	7.8	5	7.8
Important	25	10.8	4	9.1	368	35.5	86	27.7	432	41.6	48	35.0	187	30.6	8	12.5
Not very important	23	10.0	13	29.5	234	22.6	88	28.4	202	19.4	45	32.8	267	43.6	33	51.6
Not at all important	178	77.1	27	61.4	235	22.7	91	29.4	90	8.7	21	15.3	110	18.0	18	28.1
**EFFECT**																
Very important	14	6.1	3	6.7	179	17.2	34	11.0	251	24.0	22	15.9	73	12.0	2	3.1
Important	26	11.3	2	4.4	364	35.0	97	31.3	441	42.1	50	36.2	230	38.0	18	28.1
Not very important	78	33.8	7	15.6	228	21.9	90	29.0	252	24.1	36	26.1	223	36.8	25	39.1
Not at all important	113	48.9	33	73.3	268	25.8	89	28.7	103	9.8	30	21.7	80	13.2	19	29.7

Supplementary Table 1: Proportion of heavy episodic drinkers by various statements on drinking alcohol (Cont.)

	Ilorin (Nigeria)				Montevideo (Uruguay)				Moscow (Russia)				Wuhan (China)			
	Non-HED		HED		Non-HED		HED		Non-HED		HED		Non-HED		HED	
	N	%	N	%	N	%	N	%	N	%	N	%	N	%	N	%
**BAD EXAMPLES**																
Very important	42	18.2	6	13.3	347	33.3	79	25.7	265	25.4	18	14.0	91	14.9	5	7.8
Important	46	19.9	2	4.4	336	32.3	104	33.9	412	39.4	47	36.4	283	46.5	19	29.7
Not very important	51	22.1	13	28.9	158	15.2	54	17.6	220	21.1	37	28.7	142	23.3	26	40.6
Not at all important	92	39.8	24	53.3	200	19.2	70	22.8	148	14.2	27	20.9	93	15.3	14	21.9
**PREVIOUSLY HURT BY OTHERS DRINKING**																
Very important	12	5.2	-	-	222	21.3	45	14.5	227	23.0	12	9.8	37	6.4	1	1.7
Important	18	7.8	1	2.3	187	17.9	75	24.2	301	30.4	37	30.3	138	24.0	13	22.0
Not very important	59	25.7	13	29.5	205	19.7	55	17.7	241	24.4	36	29.5	205	35.6	25	42.4
Not at all important	141	61.3	30	68.2	428	41.1	135	43.5	220	22.2	37	30.3	196	34.0	20	33.9
**WORK/SCHOOL**																
Very important	23	10.0	5	11.4	284	27.2	62	20.1	384	37.3	27	20.8	92	15.0	12	19.4
Important	39	17.0	6	13.6	444	42.5	131	42.4	369	35.8	46	35.4	296	48.3	25	40.3
Not very important	60	26.1	6	13.6	118	11.3	51	16.5	160	15.5	34	26.2	140	22.8	13	21.0
Not at all important	108	47.0	27	61.4	198	19.0	65	21.0	117	11.4	23	17.7	85	13.9	12	19.4
**COST**																
Very important	6	2.6	-	-	131	12.6	17	5.5	240	23.5	24	18.2	26	4.3	3	4.8
Important	17	7.4	1	2.3	327	31.4	103	33.2	338	33.1	41	31.1	93	15.3	6	9.5
Not very important	75	32.5	8	18.2	254	24.4	80	25.8	291	28.5	36	27.3	305	50.3	28	44.4
Not at all important	133	57.6	35	79.5	329	31.6	110	35.5	153	15.0	31	23.5	182	30.0	26	41.3

Supplementary Table 1: Proportion of heavy episodic drinkers by various statements on drinking alcohol (Cont.)

	Ilorin (Nigeria)				Montevideo (Uruguay)				Moscow (Russia)				Wuhan (China)			
	Non-HED		HED		Non-HED		HED		Non-HED		HED		Non-HED		HED	
	N	%	N	%	N	%	N	%	N	%	N	%	N	%	N	%
**RELIGION**																
Very important	79	34.2	6	13.6	29	2.8	4	1.3	103	10.7	6	4.7	24	4.2	3	5.3
Important	62	26.8	13	29.5	47	4.5	15	4.8	200	20.7	18	14.0	67	11.6	12	21.1
Not very important	26	11.3	9	20.5	170	16.4	56	18.0	260	26.9	35	27.1	204	35.3	20	35.1
Not at all important	64	27.7	16	36.4	792	76.3	236	75.9	404	41.8	70	54.3	283	49.0	22	38.6
**UPBRINGING**																
Very important	40	17.2	5	11.4	63	6.1	9	2.9	179	17.7	10	7.9	54	8.8	4	6.2
Important	57	24.6	4	9.1	205	19.7	60	19.3	423	41.8	37	29.4	188	30.8	18	27.7
Not very important	44	19.0	7	15.9	264	25.4	71	22.8	255	25.2	48	38.1	220	36.0	27	41.5
Not at all important	91	39.2	28	63.6	506	48.7	171	55.0	154	15.2	31	24.6	149	24.4	16	24.6
**ALCOHOLISM**																
Very important	9	3.9	1	2.3	59	5.7	12	3.9	126	12.6	10	7.8	66	11.1	4	6.2
Important	19	8.2	1	2.3	112	10.7	36	11.7	190	19.0	20	15.5	181	30.3	16	24.6
Not very important	39	16.9	6	13.6	160	15.3	46	14.9	210	21.0	55	42.6	164	27.5	24	36.9
Not at all important	164	71.0	36	81.8	712	68.3	215	69.6	474	47.4	44	34.1	186	31.2	21	32.3
**TOO YOUNG**																
Very important	4	1.7	3	6.7	63	6.1	9	2.9	178	17.4	8	5.9	72	11.7	3	4.7
Important	27	11.7	1	2.2	201	19.3	59	19.0	272	26.6	25	18.5	176	28.6	19	29.7
Not very important	41	17.7	6	13.3	221	21.2	57	18.4	287	28.1	64	47.4	211	34.3	27	42.2
Not at all important	159	68.8	35	77.8	556	53.4	185	59.7	285	27.9	38	28.1	157	25.5	15	23.4

Supplementary Table 1: Proportion of heavy episodic drinkers by various statements on drinking alcohol (Cont.)

	Ilorin (Nigeria)				Montevideo (Uruguay)				Moscow (Russia)				Wuhan (China)			
	Non-HED		HED		Non-HED		HED		Non-HED		HED		Non-HED		HED	
	N	%	N	%	N	%	N	%	N	%	N	%	N	%	N	%
**OTHERS DISAPPROVAL**																
Very important	31	13.4	6	13.6	30	2.9	2	0.6	170	16.8	14	10.7	68	11.1	3	4.7
Important	60	26.0	3	6.8	97	9.3	34	11.0	361	35.6	36	27.5	206	33.6	22	34.4
Not very important	34	14.7	5	11.4	255	24.5	58	18.7	250	24.7	47	35.9	213	34.7	23	35.9
Not at all important	106	45.9	30	68.2	660	63.3	216	69.7	232	22.9	34	26	127	20.7	16	25.0
**HEALTH**																
Very important	23	10.0	5	11.4	209	20.0	37	12.0	198	19.1	23	17.0	156	26.0	10	15.9
Important	39	16.9	16	36.4	256	24.5	74	23.9	329	31.8	39	28.9	201	33.4	22	34.9
Not very important	20	8.7	6	13.6	114	10.9	38	12.3	280	27.1	40	29.6	127	21.1	18	28.6
Not at all important	149	64.5	17	38.6	464	44.5	160	51.8	228	22.0	33	24.4	117	19.5	13	20.6
**NOT INTERESTED**																
Very important	67	29.1	7	16.3	237	22.9	28	9.1	241	23.8	13	10.0	110	18.2	2	3.2
Important	47	20.4	7	16.3	281	27.1	57	18.5	376	37.1	47	36.2	132	21.9	7	11.3
Not very important	30	13.0	3	7.0	253	24.4	77	25.0	267	26.4	40	30.8	229	38.0	35	56.5
Not at all important	86	37.4	26	60.5	266	25.7	146	47.4	129	12.7	30	23.1	132	21.9	18	29.0

Supplementary Table 1: Proportion of heavy episodic drinkers by various statements on drinking alcohol (Cont.)

	Ilorin (Nigeria)				Montevideo (Uruguay)				Moscow (Russia)				Wuhan (China)			
	Non-HED		HED		Non-HED		HED		Non-HED		HED		Non-HED		HED	
	N	%	N	%	N	%	N	%	N	%	N	%	N	%	N	%
Agree or disagree with the following statements																
**DRINKING AS A PLEASURE OF LIFE**																
Strongly agree	77	33.2	21	47.7	67	6.1	53	17.0	64	6.0	19	13.7	98	14.4	7	10.8
Agree	113	48.7	18	40.9	280	25.6	92	29.6	237	22.3	56	40.3	217	31.8	30	46.2
Neither agree nor disagree	21	9.1	-	-	282	25.8	70	22.5	334	31.4	43	30.9	209	30.6	18	27.7
Disagree	12	5.2	2	4.5	367	33.6	87	28.0	227	21.3	16	11.5	126	18.5	8	12.3
Strongly disagree	9	3.9	3	6.8	96	8.8	9	2.9	202	19.0	5	3.6	32	4.7	2	3.1
**DRINKING AS BEING FRIENDLY**																
Strongly agree	73	30.8	20	45.5	37	3.4	31	10.0	61	5.7	13	9.3	132	19.4	8	12.5
Agree	128	54.0	18	40.9	411	37.6	137	44.2	292	27.4	60	42.9	383	56.3	45	70.3
Neither agree nor disagree	17	7.2	2	4.5	248	22.7	61	19.7	343	32.1	46	32.9	118	17.4	10	15.6
Disagree	11	4.6	-	-	335	30.6	70	22.6	216	20.2	19	13.6	44	6.5	1	1.6
Strongly disagree	8	3.4	4	9.1	62	5.7	11	3.5	155	14.5	2	1.4	3	0.4	-	-

Supplementary Table 1: Proportion of heavy episodic drinkers by various statements on drinking alcohol (Cont.)

	Ilorin (Nigeria)				Montevideo (Uruguay)				Moscow (Russia)				Wuhan (China)			
	Non-HED		HED		Non-HED		HED		Non-HED		HED		Non-HED		HED	
	N	%	N	%	N	%	N	%	N	%	N	%	N	%	N	%
**NOTHING GOOD ABOUT DRINKING**																
Strongly agree	21	8.9	9	20.5	64	5.9	4	1.3	120	11.3	12	8.7	107	15.8	5	7.8
Agree	9	3.8	2	4.5	239	21.9	47	15.1	192	18.1	13	9.4	169	24.9	23	35.9
Neither agree nor disagree	40	17.0	1	2.3	374	34.2	97	31.2	451	42.5	70	50.7	242	35.7	12	18.8
Disagree	121	51.5	21	47.7	366	33.5	131	42.1	219	20.7	33	23.9	156	23.0	22	34.4
Strongly disagree	44	18.7	11	25.0	50	4.6	32	10.3	78	7.4	10	7.2	4	0.6	2	3.1
Opinion of a person in the following situations on how much they should drink **MOTHER WITH SMALL CHILDREN**																
0 drinks	208	88.5	43	97.7	931	85.3	228	73.8	978	90.4	111	81.6	413	62.3	35	60.3
Some drinking but not enough to feel the effects (1 or 2 drinks)	27	11.5	1	2.3	152	13.9	80	25.9	92	8.5	22	16.2	208	31.4	22	37.9
Enough to feel the effects but not become drunk	-	-	-	-	8	0.7	-	-	6	0.6	2	1.5	36	5.4	1	1.7
Getting drunk is sometimes alright	-	-	-	-	1	0.1	1	0.3	3	0.3	-	-	6	0.9	-	-
Getting drunk is always alright	-	-	-	-	-	-	-	-	3	0.3	1	0.7	-	-	-	-

Supplementary Table 1: Proportion of heavy episodic drinkers by various statements on drinking alcohol (Cont.)

	Ilorin (Nigeria)				Montevideo (Uruguay)				Moscow (Russia)				Wuhan (China)			
	Non-HED		HED		Non-HED		HED		Non-HED		HED		Non-HED		HED	
	N	%	N	%	N	%	N	%	N	%	N	%	N	%	N	%
**FATHER WITH SMALL CHILDREN**																
0 drinks	153	64.6	27	62.8	920	84.4	224	72.5	854	79.6	71	51.4	346	52.0	23	35.4
Some drinking but not enough to feel the effects (1 or 2 drinks)	69	29.1	9	20.9	162	14.9	80	25.9	197	18.4	51	37	246	37.0	34	52.3
Enough to feel the effects but not become drunk	15	6.3	7	16.3	8	0.7	4	1.3	18	1.7	9	6.5	58	8.7	8	12.3
Getting drunk is sometimes alright	-	-	-	-	-	-	1	0.3	1	0.1	3	2.2	15	2.3	0	0
Getting drunk is always alright	-	-	-	-	-	-	-	-	3	0.3	4	2.9	-	-	-	-
**MAN AT BAR WITH FRIENDS**																
0 drinks	33	14.0	-	-	52	4.8	10	3.2	29	2.7	1	0.7	30	4.5	-	-
Some drinking but not enough to feel the effects (1 or 2 drinks)	114	48.5	19	47.5	593	54.7	121	38.9	277	25.9	18	13.0	433	64.4	35	53.8
Enough to feel the effects but not become drunk	70	29.8	20	50.0	340	31.3	120	38.6	492	45.9	64	46.4	164	24.4	26	40.0
Getting drunk is sometimes alright	17	7.2	1	2.5	89	8.2	56	18.0	213	19.9	47	34.1	42	6.3	4	6.2
Getting drunk is always alright	1	0.4	-	-	11	1.0	4	1.3	60	5.6	8	5.8	3	0.4	-	-

Supplementary Table 1: Proportion of heavy episodic drinkers by various statements on drinking alcohol (Cont.)

	Ilorin (Nigeria)				Montevideo (Uruguay)				Moscow (Russia)				Wuhan (China)			
	Non-HED		HED		Non-HED		HED		Non-HED		HED		Non-HED		HED	
	N	%	N	%	N	%	N	%	N	%	N	%	N	%	N	%
**WOMAN AT BAR WITH FRIENDS**																
0 drinks	110	46.8	25	61.0	102	9.4	21	6.8	105	9.8	16	11.5	93	14.3	7	12.5
Some drinking but not enough to feel the effects (1 or 2 drinks)	102	43.4	11	26.8	581	53.4	134	43.2	515	48.1	42	30.2	419	64.5	31	55.4
Enough to feel the effects but not become drunk	19	8.1	5	12.2	322	29.6	100	32.3	352	32.9	62	44.6	122	18.8	17	30.4
Getting drunk is sometimes alright	4	1.7	-	-	74	6.8	54	17.4	83	7.8	15	10.8	16	2.5	1	1.8
Getting drunk is always alright	-	-	-	-	9	0.8	1	0.3	15	1.4	4	2.9	-	-	-	-
**WOMAN OUT WITH CO-WORKERS**																
0 drinks	124	52.8	30	71.4	113	10.4	13	4.2	131	12.3	23	16.9	36	5.4	-	-
Some drinking but not enough to feel the effects (1 or 2 drinks)	93	39.6	7	16.7	587	53.9	115	37.2	574	54.1	43	31.6	406	60.4	33	51.6
Enough to feel the effects but not become drunk	14	6.0	5	11.9	324	29.8	123	39.8	320	30.2	59	43.4	187	27.8	27	42.2
Getting drunk is sometimes alright	4	1.7	-	-	57	5.2	52	16.8	32	3.0	8	5.9	41	6.1	2	3.1
Getting drunk is always alright	-	-	-	-	8	0.7	6	1.9	4	0.4	3	2.2	2	0.3	2	3.1

Supplementary Table 1: Proportion of heavy episodic drinkers by various statements on drinking alcohol (Cont.)

	Ilorin (Nigeria)				Montevideo (Uruguay)				Moscow (Russia)				Wuhan (China)			
	Non-HED		HED		Non-HED		HED		Non-HED		HED		Non-HED		HED	
	N	%	N	%	N	%	N	%	N	%	N	%	N	%	N	%
**MAN OUT WITH CO-WORKERS**																
0 drinks	43	18.2	10	25.0	143	13.1	16	5.2	42	3.9	2	1.5	130	19.9	13	23.6
Some drinking but not enough to feel the effects (1 or 2 drinks)	120	50.8	21	52.5	585	53.8	129	41.6	349	32.6	18	13.2	382	58.5	26	47.3
Enough to feel the effects but not become drunk	57	24.2	9	22.5	302	27.8	115	37.1	488	45.6	77	56.6	132	20.2	14	25.5
Getting drunk is sometimes alright	15	6.4	-	-	53	4.9	49	15.8	173	16.2	32	23.5	8	1.2	2	3.6
Getting drunk is always alright	1	0.4	-	-	5	0.5	1	0.3	18	1.7	7	5.1	1	0.2	-	-
**MAN HAVING DINNER WITH PARTNER**																
0 drinks	84	36.1	14	33.3	102	9.4	22	7.1	54	5.1	5	3.6	52	7.7	4	6.6
Some drinking but not enough to feel the effects (1 or 2 drinks)	123	52.8	25	59.5	681	62.5	181	58.4	559	52.9	51	37.2	444	66.0	34	55.7
Enough to feel the effects but not become drunk	24	10.3	3	7.1	214	19.7	71	22.9	348	32.9	57	41.6	127	18.9	13	21.3
Getting drunk is sometimes alright	2	0.9	-	-	86	7.9	31	10.0	76	7.2	16	11.7	38	5.6	8	13.1
Getting drunk is always alright	-	-	-	-	6	0.6	5	1.6	20	1.9	8	5.8	12	1.8	2	3.3

Supplementary Table 1: Proportion of heavy episodic drinkers by various statements on drinking alcohol (Cont.)

	Ilorin (Nigeria)				Montevideo (Uruguay)				Moscow (Russia)				Wuhan (China)			
	Non-HED		HED		Non-HED		HED		Non-HED		HED		Non-HED		HED	
	N	%	N	%	N	%	N	%	N	%	N	%	N	%	N	%
**WOMAN HAVING DINNER WITH PARTNER**																
0 drinks	124	52.8	20	50.0	101	9.3	26	8.4	113	10.8	14	10.2	66	10.2	5	8.8
Some drinking but not enough to feel the effects (1 or 2 drinks)	102	43.4	19	47.5	686	63.0	175	56.5	661	63.0	67	48.9	420	64.8	32	56.1
Enough to feel the effects but not become drunk	8	3.4	1	2.5	213	19.6	71	22.9	241	23.0	42	30.7	126	19.4	12	21.1
Getting drunk is sometimes alright	1	0.4	-	-	83	7.6	34	11.0	26	2.5	10	7.3	27	4.2	7	12.3
Getting drunk is always alright	-	-	-	-	6	0.6	4	1.3	8	0.8	4	2.9	9	1.4	1	1.8

Supplementary Table 2: Proportion of heavy episodic drinkers by effects of drinking alcohol

	Ilorin (Nigeria)		Montevideo (Uruguay)		Moscow (Russia)		Wuhan (China)	
	N	%	N	%	N	%	N	%
**Relaxed**								
Very often true	22	50.0	76	24.4	65	46.4	8	12.5
Often true	16	36.4	84	27.0	56	40.0	26	40.6
Sometimes true	4	9.1	88	28.3	12	8.6	22	34.4
Rarely true	1	2.3	35	11.3	7	5.0	4	6.3
Never true	1	2.3	28	9.0	-	-	4	6.3
**Happy**								
Very often true	17	38.6	88	28.4	47	34.8	48	7.8
Often true	17	38.6	92	29.7	40	29.6	216	35.1
Sometimes true	5	11.4	71	22.9	32	23.7	217	35.3
Rarely true	4	9.1	29	9.4	11	8.1	81	13.2
Never true	1	2.3	30	9.7	5	3.7	53	8.6
**Aggressive**								
Very often true	-	-	14	4.5	7	5.1	-	-
Often true	3	6.8	12	3.9	14	10.3	1	1.6
Sometimes true	3	6.8	22	7.1	34	25.0	7	11.5
Rarely true	4	9.1	54	17.4	41	30.1	27	44.3
Never true	34	77.3	208	67.1	40	29.4	26	42.6
**Friendly**								
Very often true	7	15.9	45	14.6	34	24.8	3	4.8
Often true	10	22.7	74	24.0	51	37.2	26	41.3
Sometimes true	9	20.5	100	32.5	41	29.9	12	19.0
Rarely true	10	22.7	44	14.3	7	5.1	13	20.6
Never true	8	18.2	45	14.6	4	2.9	9	14.3
**Easier to talk**								
Very often true	9	20.0	41	13.2	38	27.7	2	3.1
Often true	9	20.0	54	17.4	56	40.9	22	34.4
Sometimes true	10	22.2	73	23.5	32	23.4	17	26.6
Rarely true	4	8.9	54	17.4	9	6.6	15	23.4
Never true	13	28.9	88	28.4	2	1.5	8	12.5
**Forget problems**								
Very often true	6	14.0	27	8.7	45	32.6	1	1.5
Often true	7	16.3	58	18.6	43	31.2	20	30.8
Sometimes true	9	20.9	58	18.6	32	23.2	20	30.8
Rarely true	7	16.3	45	14.5	13	9.4	14	21.5
Never true	14	32.6	123	39.5	5	3.6	10	15.4
**Regret**								
Very often true	-	-	7	2.3	8	5.9	-	-
Often true	1	2.3	19	6.1	21	15.4	2	3.3
Sometimes true	7	15.9	55	17.7	45	33.1	8	13.3
Rarely true	1	2.3	53	17.1	38	27.9	18	30.0
Never true	35	79.5	176	56.8	24	17.6	32	53.3
**Sex more pleasurable**								
Very often true	7	15.9	27	8.8	18	14.9	1	2.0
Often true	7	15.9	29	9.4	37	30.6	4	8.0
Sometimes true	11	25.0	81	26.3	28	23.1	12	24.0
Rarely true	3	6.8	40	13.0	22	18.2	12	24.0
Never true	16	36.4	131	42.5	16	13.2	21	42.0

Supplementary Table 2: Proportion of heavy episodic drinkers by effects of drinking alcohol (Cont.)

	Ilorin (Nigeria)		Montevideo (Uruguay)		Moscow (Russia)		Wuhan (China)	
	N	%	N	%	N	%	N	%
**Feel more attractive**								
Very often true	8	18.6	14	4.5	32	25.0	1	1.9
Often true	9	20.9	19	6.2	23	18.0	3	5.8
Sometimes true	10	23.3	61	19.8	41	32.0	8	15.4
Rarely true	4	9.3	47	15.3	20	15.6	11	21.2
Never true	12	27.9	167	54.2	12	9.4	29	55.8
**Trouble with police**								
Very often true	-	-	4	1.3	-	-	-	-
Often true	-	-	5	1.6	-	-	-	-
Sometimes true	-	-	17	5.5	19	13.9	1	1.8
Rarely true	5	11.4	25	8.1	27	19.7	8	14.0
Never true	39	88.6	259	83.5	91	66.4	48	84.2
**Fun**								
Very often true	11	24.4	153	49.7	43	30.9	1	1.6
Often true	10	22.2	94	30.5	52	37.4	14	22.2
Sometimes true	13	28.9	46	14.9	33	23.7	16	25.4
Rarely true	4	8.9	8	2.6	6	4.3	18	28.6
Never true	7	15.6	7	2.3	5	3.6	14	22.2
**Black out**								
Very often true	-	-	5	1.6	1	0.7	-	-
Often true	-	-	13	4.2	15	10.9	3	4.7
Sometimes true	3	7.0	54	17.4	27	19.7	13	20.3
Rarely true	6	14.0	65	20.9	52	38.0	24	37.5
Never true	-	-	5	1.6	1	0.7	-	-

Supplementary Table 3: Proportion of heavy episodic drinkers by importance of the following reasons for drinking alcohol

	Ilorin (Nigeria)		Montevideo (Uruguay)		Moscow (Russia)		Wuhan (China)	
	N	%	N	%	N	%	N	%
**Health**								
Very important	4	9.1	9	2.9	19	14.2	2	3.2
Important	13	29.5	14	4.5	34	25.4	7	11.1
Not very important	10	22.7	43	14.0	44	32.8	36	57.1
Not at all important	17	38.6	242	78.6	37	27.6	18	28.6
**Feel good**								
Very important	15	34.1	27	8.7	23	17.0	5	7.9
Important	23	52.3	107	34.6	63	46.7	16	25.4
Not very important	5	11.4	88	28.5	30	22.2	34	54.0
Not at all important	1	2.3	87	28.2	19	14.1	8	12.7
**Forget worries**								
Very important	5	11.4	12	3.9	43	31.4	2	3.1
Important	14	31.8	90	29.0	61	44.5	18	28.1
Not very important	8	18.2	73	23.5	26	19.0	31	48.4
Not at all important	17	38.6	135	43.5	7	5.1	13	20.3
**Inhibition**								
Very important	4	9.3	22	7.2	27	19.6	3	4.6
Important	3	7.0	83	27.0	58	42.0	9	13.8
Not very important	14	32.6	76	24.8	36	26.1	37	56.9
Not at all important	22	51.2	126	41.0	17	12.3	16	24.6
**Celebrate**								
Very important	26	57.8	162	52.4	68	49.6	15	23.4
Important	15	33.3	121	39.2	49	35.8	36	56.3
Not very important	1	2.2	13	4.2	16	11.7	10	15.6
Not at all important	3	6.7	13	4.2	4	2.9	3	4.7
**Taste**								
Very important	3	6.8	57	18.4	44	32.4	-	-
Important	4	9.1	153	49.4	47	34.6	6	9.4
Not very important	10	22.7	49	15.8	36	26.5	30	46.9
Not at all important	27	61.4	51	16.5	9	6.6	28	43.8
**Thirst**								
Very important	-	-	18	5.8	19	13.9	1	1.6
Important	6	14.0	61	19.7	47	34.3	1	1.6
Not very important	8	18.6	66	21.4	43	31.4	18	28.1
Not at all important	29	67.4	164	53.1	28	20.4	44	68.8

Supplementary Table 4: Proportion of heavy episodic drinkers by importance of the following reasons for limiting or not drinking alcohol at all

	Ilorin (Nigeria)		Montevideo (Uruguay)		Moscow (Russia)		Wuhan (China)	
	N	%	N	%	N	%	N	%
**Pregnant**								
Very important	-	-	47	64.4	20	71.4	-	-
Important	-	-	12	16.4	5	17.9	-	-
Not very important	4	36.4	8	11.0	1	3.6	-	-
Not at all important	7	63.6	6	8.2	2	7.1	-	-
**Taste**								
Very important	-	-	45	14.5	23	16.8	5	7.8
Important	4	9.1	86	27.7	48	35.0	8	12.5
Not very important	13	29.5	88	28.4	45	32.8	33	51.6
Not at all important	27	61.4	91	29.4	21	15.3	18	28.1
**Effect**								
Very important	3	6.7	34	11.0	22	15.9	2	3.1
Important	2	4.4	97	31.3	50	36.2	18	28.1
Not very important	7	15.6	90	29.0	36	26.1	25	39.1
Not at all important	33	73.3	89	28.7	30	21.7	19	29.7
**Bad examples**								
Very important	6	13.3	79	25.7	18	14.0	5	7.8
Important	2	4.4	104	33.9	47	36.4	19	29.7
Not very important	13	28.9	54	17.6	37	28.7	26	40.6
Not at all important	24	53.3	70	22.8	27	20.9	14	21.9
**Previously hurt by others drinking**	-	-	45	14.5	12	9.8	1	1.7
Very important	1	2.3	75	24.2	37	30.3	13	22.0
Important	13	29.5	55	17.7	36	29.5	25	42.4
Not very important	30	68.2	135	43.5	37	30.3	20	33.9
Not at all important	-	-	45	14.5	12	9.8	1	1.7
**Work/school**								
Very important	5	11.4	62	20.1	27	20.8	12	19.4
Important	6	13.6	131	42.4	46	35.4	25	40.3
Not very important	6	13.6	51	16.5	34	26.2	13	21.0
Not at all important	27	61.4	65	21.0	23	17.7	12	19.4
**Cost**								
Very important	-	-	17	5.5	24	18.2	3	4.8
Important	1	2.3	103	33.2	41	31.1	6	9.5
Not very important	8	18.2	80	25.8	36	27.3	28	44.4
Not at all important	35	79.5	110	35.5	31	23.5	26	41.3
**Religion**								
Very important	6	13.6	4	1.3	6	4.7	3	5.3
Important	13	29.5	15	4.8	18	14.0	12	21.1
Not very important	9	20.5	56	18.0	35	27.1	20	35.1
Not at all important	16	36.4	236	75.9	70	54.3	22	38.6
**Upbringing**								
Very important	5	11.4	9	2.9	10	7.9	4	6.2
Important	4	9.1	60	19.3	37	29.4	18	27.7
Not very important	7	15.9	71	22.8	48	38.1	27	41.5
Not at all important	28	63.6	171	55.0	31	24.6	16	24.6

Supplementary Table 4: Proportion of heavy episodic drinkers by importance of the following reasons for limiting or not drinking alcohol at all (Cont.)

	Ilorin (Nigeria)		Montevideo (Uruguay)		Moscow (Russia)		Wuhan (China)	
	N	%	N	%	N	%	N	%
**Alcoholism**								
Very important	1	2.3	12	3.9	10	7.8	4	6.2
Important	1	2.3	36	11.7	20	15.5	16	24.6
Not very important	6	13.6	46	14.9	55	42.6	24	36.9
Not at all important	36	81.8	215	69.6	44	34.1	21	32.3
**Too young**								
Very important	3	6.7	9	2.9	8	5.9	3	4.7
Important	1	2.2	59	19.0	25	18.5	19	29.7
Not very important	6	13.3	57	18.4	64	47.4	27	42.2
Not at all important	35	77.8	185	59.7	38	28.1	15	23.4
**Others disapproval**								
Very important	6	13.6	2	0.6	14	10.7	3	4.7
Important	3	6.8	34	11.0	36	27.5	22	34.4
Not very important	5	11.4	58	18.7	47	35.9	23	35.9
Not at all important	30	68.2	216	69.7	34	26.0	16	25.0
**Health**								
Very important	5	11.4	37	12.0	23	17	10	15.9
Important	16	36.4	74	23.9	39	28.9	22	34.9
Not very important	6	13.6	38	12.3	40	29.6	18	28.6
Not at all important	17	38.6	160	51.8	33	24.4	13	20.6
**Not interested**								
Very important	7	16.3	28	9.1	13	10.0	2	3.2
Important	7	16.3	57	18.5	47	36.2	7	11.3
Not very important	3	7.0	77	25.0	40	30.8	35	56.5
Not at all important	26	60.5	146	47.4	30	23.1	18	29.0

Supplementary Table 5: Proportion of heavy episodic drinkers by statements on alcohol

	Ilorin (Nigeria)		Montevideo (Uruguay)		Moscow (Russia)		Wuhan (China)	
	**N**	**%**	**N**	**%**	**N**	**%**	**N**	**%**
**Drinking as a pleasure of life**								
Strongly agree	21	47.7	53	17.0	19	13.7	7	10.8
Agree	18	40.9	92	29.6	56	40.3	30	46.2
Neither agree nor disagree	-	-	70	22.5	43	30.9	18	27.7
Disagree	2	4.5	87	28.0	16	11.5	8	12.3
Strongly disagree	3	6.8	9	2.9	5	3.6	2	3.1
**Drinking as being friendly**								
Strongly agree	20	45.5	31	10.0	13	9.3	8	12.5
Agree	18	40.9	137	44.2	60	42.9	45	70.3
Neither agree nor disagree	2	4.5	61	19.7	46	32.9	10	15.6
Disagree	-	-	70	22.6	19	13.6	1	1.6
Strongly disagree	4	9.1	11	3.5	2	1.4	-	-
**Nothing good about drinking**								
Strongly agree	9	20.5	4	1.3	12	8.7	5	7.8
Agree	2	4.5	47	15.1	13	9.4	23	35.9
Neither agree nor disagree	1	2.3	97	31.2	70	50.7	12	18.8
Disagree	21	47.7	131	42.1	33	23.9	22	34.4
Strongly disagree	11	25.0	32	10.3	10	7.2	2	3.1

Supplementary Table 6: Proportion of heavy episodic drinkers by opinion of a person in the following situations on how much they should drink

	Ilorin (Nigeria)		Montevideo (Uruguay)		Moscow (Russia)		Wuhan (China)	
	N	%	N	%	N	%	N	%
**Mother with small children**								
0 drinks	43	97.7	228	73.8	111	81.6	35	60.3
Some drinking but not enough to feel the effects (1 or 2 drinks)	1	2.3	80	25.9	22	16.2	22	37.9
Enough to feel the effects but not become drunk	-	-	-	-	2	1.5	1	1.7
Getting drunk is sometimes alright	-	-	1	0.3	-	-	-	-
Getting drunk is always alright	-	-	-	-	1	0.7	-	-
**Father with small children**								
0 drinks	27	62.8	224	72.5	71	51.4	23	35.4
Some drinking but not enough to feel the effects (1 or 2 drinks)	9	20.9	80	25.9	51	37.0	34	52.3
Enough to feel the effects but not become drunk	7	16.3	4	1.3	9	6.5	8	12.3
Getting drunk is sometimes alright	-	-	1	0.3	3	2.2	-	-
Getting drunk is always alright	-	-	-	-	4	2.9	-	-
**Man at bar with friends**								
0 drinks	-	-	10	3.2	1	0.7	-	-
Some drinking but not enough to feel the effects (1 or 2 drinks)	19	47.5	121	38.9	18	13.0	35	53.8
Enough to feel the effects but not become drunk	20	50.0	120	38.6	64	46.4	26	40.0
Getting drunk is sometimes alright	1	2.5	56	18.0	47	34.1	4	6.2
Getting drunk is always alright	-	-	4	1.3	8	5.8	-	-
**Woman at bar with friends**								
0 drinks	25	61.0	21	6.8	16	11.5	7	12.5
Some drinking but not enough to feel the effects (1 or 2 drinks)	11	26.8	134	43.2	42	30.2	31	55.4
Enough to feel the effects but not become drunk	5	12.2	100	32.3	62	44.6	17	30.4
Getting drunk is sometimes alright	-	-	54	17.4	15	10.8	1	1.8
Getting drunk is always alright	-	-	1	0.3	4	2.9	-	-
**Woman out with co-workers**								
0 drinks	30	71.4	13	4.2	23	16.9	-	-
Some drinking but not enough to feel the effects (1 or 2 drinks)	7	16.7	115	37.2	43	31.6	33	51.6
Enough to feel the effects but not become drunk	5	11.9	123	39.8	59	43.4	27	42.2
Getting drunk is sometimes alright	-	-	52	16.8	8	5.9	2	3.1
Getting drunk is always alright	-	-	6	1.9	3	2.2	2	3.1

Supplementary Table 6: Proportion of heavy episodic drinkers by opinion of a person in the following situations on how much they should drink (Cont.)

	Ilorin (Nigeria)		Montevideo (Uruguay)		Moscow (Russia)		Wuhan (China)	
	N	%	N	%	N	%	N	%
**Man out with co-workers**								
0 drinks	10	25.0	16	5.2	2	1.5	13	23.6
Some drinking but not enough to feel the effects (1 or 2 drinks)	21	52.5	129	41.6	18	13.2	26	47.3
Enough to feel the effects but not become drunk	9	22.5	115	37.1	77	56.6	14	25.5
Getting drunk is sometimes alright	-	-	49	15.8	32	23.5	2	3.6
Getting drunk is always alright	-	-	1	0.3	7	5.1	-	-
**Man having dinner with partner**								
0 drinks	14	33.3	22	7.1	5	3.6	4	6.6
Some drinking but not enough to feel the effects (1 or 2 drinks)	25	59.5	181	58.4	51	37.2	34	55.7
Enough to feel the effects but not become drunk	3	7.1	71	22.9	57	41.6	13	21.3
Getting drunk is sometimes alright	-	-	31	10.0	16	11.7	8	13.1
Getting drunk is always alright	-	-	5	1.6	8	5.8	2	3.3
**Woman having dinner with partner**								
0 drinks	20	50.0	26	8.4	14	10.2	5	8.8
Some drinking but not enough to feel the effects (1 or 2 drinks)	19	47.5	175	56.5	67	48.9	32	56.1
Enough to feel the effects but not become drunk	1	2.5	71	22.9	42	30.7	12	21.1
Getting drunk is sometimes alright	-	-	34	11.0	10	7.3	7	12.3
Getting drunk is always alright	-	-	4	1.3	4	2.9	1	1.8
